# Anti-tumor activity of high-dose EGFR tyrosine kinase inhibitor and sequential docetaxel in wild type EGFR non-small cell lung cancer cell nude mouse xenografts

**DOI:** 10.18632/oncotarget.13327

**Published:** 2016-11-12

**Authors:** Ning Tang, Qianqian Zhang, Shu Fang, Xiao Han, Zhehai Wang

**Affiliations:** ^1^ Department of Oncology, Shandong Cancer Hospital and Institute, Jinan, China; ^2^ Department of Jining Number One People's Hospital, Jinan, China

**Keywords:** non-small cell lung cancer, epidermal growth factor receptor (EGFR), icotinib, docetaxel, nude mouse xenografts

## Abstract

Treatment of non-small-cell lung cancer (NSCLC) with wild-type epidermal growth factor receptor (EGFR) is still a challenge. This study explored antitumor activity of high-dose icotinib (an EGFR tyrosine kinase inhibitor) plus sequential docetaxel against wild-type EGFR NSCLC cells-generated nude mouse xenografts. Nude mice were subcutaneously injected with wild-type EGFR NSCLC A549 cells and divided into different groups for 3-week treatment. Tumor xenograft volumes were monitored and recorded, and at the end of experiments, tumor xenografts were removed for Western blot and immunohistochemical analyses. Compared to control groups (negative control, regular-dose icotinib [IcoR], high-dose icotinib [IcoH], and docetaxel [DTX]) and regular icotinib dose (60 mg/kg) with docetaxel, treatment of mice with a high-dose (1200 mg/kg) of icotinib plus sequential docetaxel for 3 weeks (IcoH-DTX) had an additive effect on suppression of tumor xenograft size and volume (*P* < 0.05). Icotinib-containing treatments markedly reduced phosphorylation of EGFR, mitogen activated protein kinase (MAPK), and protein kinase B (Akt), but only the high-dose icotinib-containing treatments showed an additive effect on CD34 inhibition (*P* < 0.05), an indication of reduced microvessel density in tumor xenografts. Moreover, high-dose icotinib plus docetaxel had a similar effect on mouse weight loss (a common way to measure adverse reactions in mice), compared to the other treatment combinations. The study indicate that the high dose of icotinib plus sequential docetaxel (IcoH-DTX) have an additive effect on suppressing the growth of wild-type EGFR NSCLC cell nude mouse xenografts, possibly through microvessel density reduction. Future clinical trials are needed to confirm the findings of this study.

## INTRODUCTION

Lung cancer is the leading global cause of cancer-related deaths and it is estimated that more than 1.8 million new lung cancer cases and more than 1.5 million lung cancer-related deaths occurred worldwide in 2012 [1]. In China, lung cancer morbidity and mortality have continuously increased and this upward trend could continue for at least the next few decades [2]. Histologically, lung cancer can be divided into non-small cell lung cancer (NSCLC) and small cell lung cancer [3]. NSCLC comprises up to 85% of all lung cancer cases and clinically, NSCLC is frequently diagnosed at advanced stages of disease; thus, making curable surgery (tumor resection) impossible [4]. Moreover, NSCLC patients are relatively insensitive to chemo- and radiotherapy, although chemotherapy is increasingly being used neoadjuvantly and adjuvantly [5, 6].

Due to increasing research on molecular tumor markers and NCLC-driven genes, rapid development of the anti-epidermal growth factor receptor (EGFR)-targeted therapy could effectively control NSCLC progression in patients with active mutation or amplification of *EGFR*-driven tumor and increased patient survival [7–17]. For example, many randomized, open-label, phase III clinical trials have compared the effectiveness of EGFR tyrosine kinase inhibitors (EGFR-TKIs) with routine chemotherapy on NSCLC patients with mutated EGFR. The data showed that treatment response and progression-free survival of patients with EGFR-mutated NSCLC who were treated with the first-line EGFR-TKIs were significantly better than those of patients who received chemotherapy alone [7–17]. However, EGFR mutations only occur in a small proportion of NSCLC cases, i.e., 9–21% of Caucasian NSCLC patients and approximately 40% of Asian NSCLC patients [18–21]. Thus, the National Comprehensive Cancer Network (NCCN) guidelines recommend that patients with EGFR-mutated NSCLC should receive TKI treatment, while patients with wild-type EGFR NSCLC can be treated with chemotherapy [22], although chemotherapy alone cannot improve or control disease progression in the vast majority of NSCLC patients [23]. However, our recently unpublished clinical observation suggests that EGFR-TKIs plus chemotherapy was shown activity in patients with wild-type EGFR NSCLC. Thus, in this study, we explored the antitumor activity of a high-dose EGFR tyrosine kinase inhibitor (icotinib) plus the sequential chemotherapeutic drug docetaxel in wild-type EGFR NCSLC cell-generated nude mouse xenografts because icotinib and sequential docetaxel combined could have better antitumor activity and less adverse reactions than each individual drug. Icotinib hydrochloride is a novel EGFR-TKI developed in China; previous clinical trials using icotinib showed similar antitumor activity to other EGFR-TKIs in EGFR-mutated NSCLC patients [24–28] and it was subsequently approved by the Chinese State Food and Drug Administration in June 2011. In this study we investigated the efficacy and safety of icotinib plus sequential docetaxel (alone and in combination) against wild-type *EGFR* NSCLC in an animal model to determine whether this combination treatment may potentially benefit lung cancer patients in future.

## RESULTS

### Effect of icotinib alone on suppressing growth of NSCLC cell nude mouse xenograft

We first produced a nude mouse xenograft model using a NSCLC A549 cell-line that has had a wild type of *EGFR* and EGFR expression. After tumor xenografts reached 5 to 6 mm in diameter, we treated these mice with the solvent (PBS, phosphate buffered saline), regular-dose (60 mg/kg), high-dose (1200 mg/kg) icotinib, or docetaxel (5 mg/kg) for 3 weeks (*n* = 8), which was designated as Group A experiments. We found that both regular and high doses of icotinib had a statistically significant antitumor effect in terms of the tumor growth inhibition rate (TGIR; *P* < 0.05) compared to the negative control or docetaxel treatment alone (5 mg/kg) and the latter had approximately 40% TGIR (Table 1 and Figure 1A).

**Table 1:** Tumor growth inhibition rate (TGIR) in nude mouse xenograft model (*n* = 8 mice/group, mean ± SD)

Group	3-week icotinib	3-week docetaxel	Ending tumor volume (mm^3^)	TGIR (%)	*P* value
**Group A**					
Control	Solvent (PBS)	Solvent (PBS)	920.59 ± 19.11		
IcoR	60 mg/kg on day 1,2		783.56 ± 12.50	16.93 ± 1.49	< 0.05^#,##^
IcoH	1200 mg/kg on day 1,2		658.62 ± 7.16	32.14 ± 0.86	< 0.05^#,##^
DTX		5 mg/kg on day 1	569.61 ± 10.40	43.07 ± 1.44	< 0.05^#^
**Group B**					
IcoR+DTX	60 mg/kg on day 1,2	5 mg/kg on day 1	556.83 ± 9.96	44.50 ± 1.10	< 0.05^#^
IcoR-DTX	60 mg/kg on day 1,2	5 mg/kg on day 3	572.63 ± 14.91	42.78 ± 1.77	< 0.05^#^
DTX-IcoR	60 mg/kg on day 2,3	5 mg/kg on day 1	545.31 ± 6.72	45.98 ± 0.85	< 0.05^#^
**Group C**					
IcoH+DTX	1200 mg/kg on day 1,2	5 mg/kg on day 1	438.78 ± 5.54	58.91 ± 0.71	< 0.05^#,##,▲^
IcoH-DTX	1200 mg/kg on day 1,2	5 mg/kg on day 3	374.84 ± 16.30	66.80 ± 2.05	< 0.05^#,##^
DTX-IcoH	1200 mg/kg on day 2,3	5 mg/kg on day 1	492.03 ± 7.96	52.46 ± 0.99	< 0.05^#,##,▲^

Abbreviations: Control, negative control; DTX, docetaxel; IcoR, a regular icotinib dose; IcoH, a high icotinib dose; IcoH-D, a high icotinib dose and sequential docetaxel for 3 weeks of treatment; IcoR-D, a regular icotinib dose and sequential docetaxel for 3 weeks; IcoH+D, a high icotinib dose plus docetaxel for 3 weeks; IcoR+D, a regular icotinib dose plus docetaxel for 3 weeks; D-IcoR, docetaxel and sequential regular icotinib dose for 3 weeks; D-IcoH, docetaxel and sequential high icotinib dose for 3-week treatment; TGIR, tumor growth inhibition rate.

#*P* < 0.05 compared to the negative control; ^##^*P* < 0.05 compared to docetaxel group; ▲*P* < 0.05 compared to IcoH-D.

**Figure 1:**
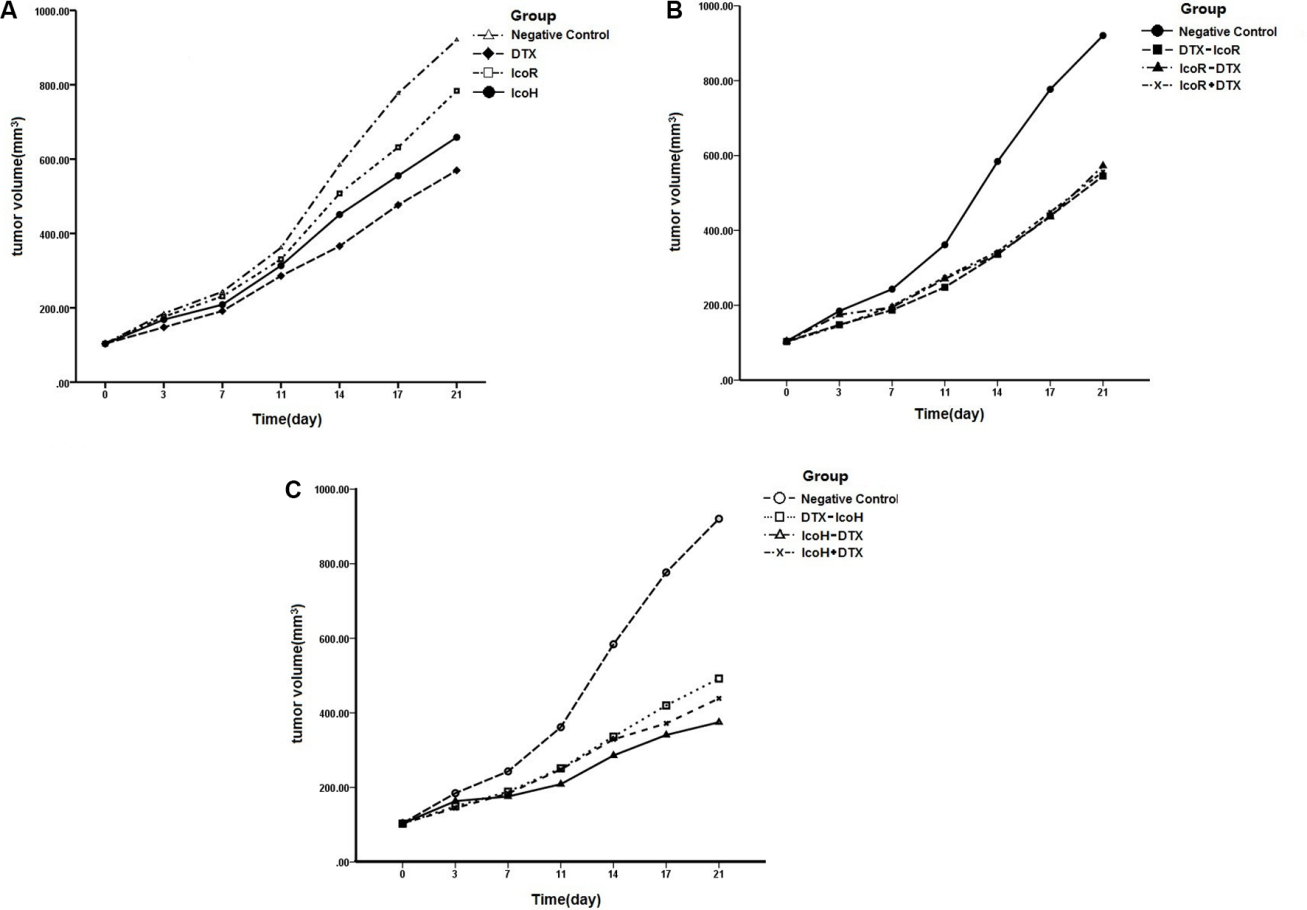
Effects of different treatments on modulation of tumor xenograft volume and size The mice were subcutaneously injected with NSCLC A549 cells and when tumor xenografts reached 5–6 mm^3^, the mice received different treatments (*n* = 8 mice/group) for up to 6 weeks. (**A**) Comparison of single drug treatment with the negative control. During the treatment, tumor xenograft volumes were measured twice a week for 3 weeks. (**B**) Comparison of a regular icotinib dose plus or sequential docetaxel treatment with the negative control. C. Comparison of a high icotinib dose plus or sequential docetaxel treatment with the negative control. Control, negative control; DTX, docetaxel; IcoR, a regular icotinib dose; IcoH, a high icotinib dose; IcoH-D, a high icotinib dose and sequential docetaxel for 3 weeks; IcoR-D, a regular icotinib dose and sequential docetaxel for 3 weeks; IcoH+D, a high icotinib dose plus docetaxel for 3 weeks; IcoR+D, a regular icotinib dose plus docetaxel for 3 weeks; D-IcoR, docetaxel and sequential regular icotinib dose for 3 weeks; D-IcoH, docetaxel and sequential high icotinib dose for 3 weeks.

### No additive effect of icotinib (60 mg/kg) plus docetaxel or sequentially with docetaxel on suppressing growth of NSCLC cell nude mouse xenografts

We then treated separate groups of mice with the regular dose of icotinib plus docetaxel (5 mg/kg) or sequential docetaxel (5 mg/kg) for 3 weeks, which was designated as Group B experiments and found there was no additive antitumor activity compared to controls in terms of TGIR (Table 1 and Figure 1B). Icotinib sequentially with docetaxel or docetaxel sequentially with icotinib showed no visible difference in TGIR compared to controls (Table 1 and Figure 1B).

### Additive effect of icotinib (1200 mg/kg) plus sequential docetaxel on suppressing growth of NSCLC cell nude mouse xenografts

We further compared the efficacy of the high-dose icotinib (1200 mg/kg) plus docetaxel and sequentially with docetaxel on suppression of NSCLC cell nude mouse xenografts, which was designated as Group C experiments. We found that an additive effect of icotinib (1200 mg/kg) plus sequential docetaxel on suppressing growth of NSCLC cell nude mouse xenografts, but there was no such an effect occurring with the high-dose icotinib (1200 mg/kg) plus docetaxel or docetaxel plus sequential icotinib (Table 1 and Figure 1C).

### Drug treatment-related toxicity in mice

During the entire experimental period of time, we monitored mouse body weight and behavior changes and found there were no statistically significant changes in body weight (Table 3).

**Table 3:** Changes in nude mouse body weight after treatment of mice with icotinib, docetaxel, or their combinations (*n* = 8 mice/group, mean ± SD)

Group	Initial body weight (g)	Ending body weight (g)	*P* value
**Group A**			
Control	20.06 ± 0.37	24.25 ± 0.51	
IcoR	20.28 ± 0.48	23.55 ± 0.24	< 0.05#,##
IcoH	20.19 ± 0.42	23.55 ± 0.32	< 0.05#,##
DTX	20.14 ± 0.34	23.08 ± 0.27	< 0.05#
**Group B**			
IcoR+DTX	19.98 ± 0.45	23.00 ± 0.40	< 0.05#
IcoR-DTX	20.25 ± 0.43	23.03 ± 0.27	< 0.05#
DTX-IcoR	20.06 ± 0.39	22.93 ± 0.32	< 0.05#
**Group C**			
IcoH+DTX	20.01 ± 0.35	22.91 ± 0.35	< 0.05#
IcoH-DTX	20.11 ± 0.43	23.00 ± 0.33	< 0.05#
DTX-IcoH	20.08 ± 0.39	23.09 ± 0.37	< 0.05#

Abbreviations: Control, negative control; DTX, docetaxel; IcoR, a regular icotinib dose; IcoH, a high icotinib dose; IcoH-D, a high icotinib dose and sequential docetaxel for 3-weeks of treatment; IcoR-D, a regular icotinib dose and sequential docetaxel for 3-weeks; IcoH+D, a high icotinib dose plus docetaxel for 3 weeks; IcoR+D, a regular icotinib dose plus docetaxel for 3 weeks; D-IcoR, docetaxel and sequential regular icotinib dose for 3 weeks; D-IcoH, docetaxel and sequential high icotinib dose for 3 weeks; TGIR, tumor growth inhibition rate.

^#^*P* < 0.05 compared to the negative control; ^##^*P* < 0.05 compared to docetaxel.

### Effect of icotinib and docetaxel treatment on regulation of EGFR, MAPK, and Akt phosphorylation in tumor cell xenografts

**Figure 2:**
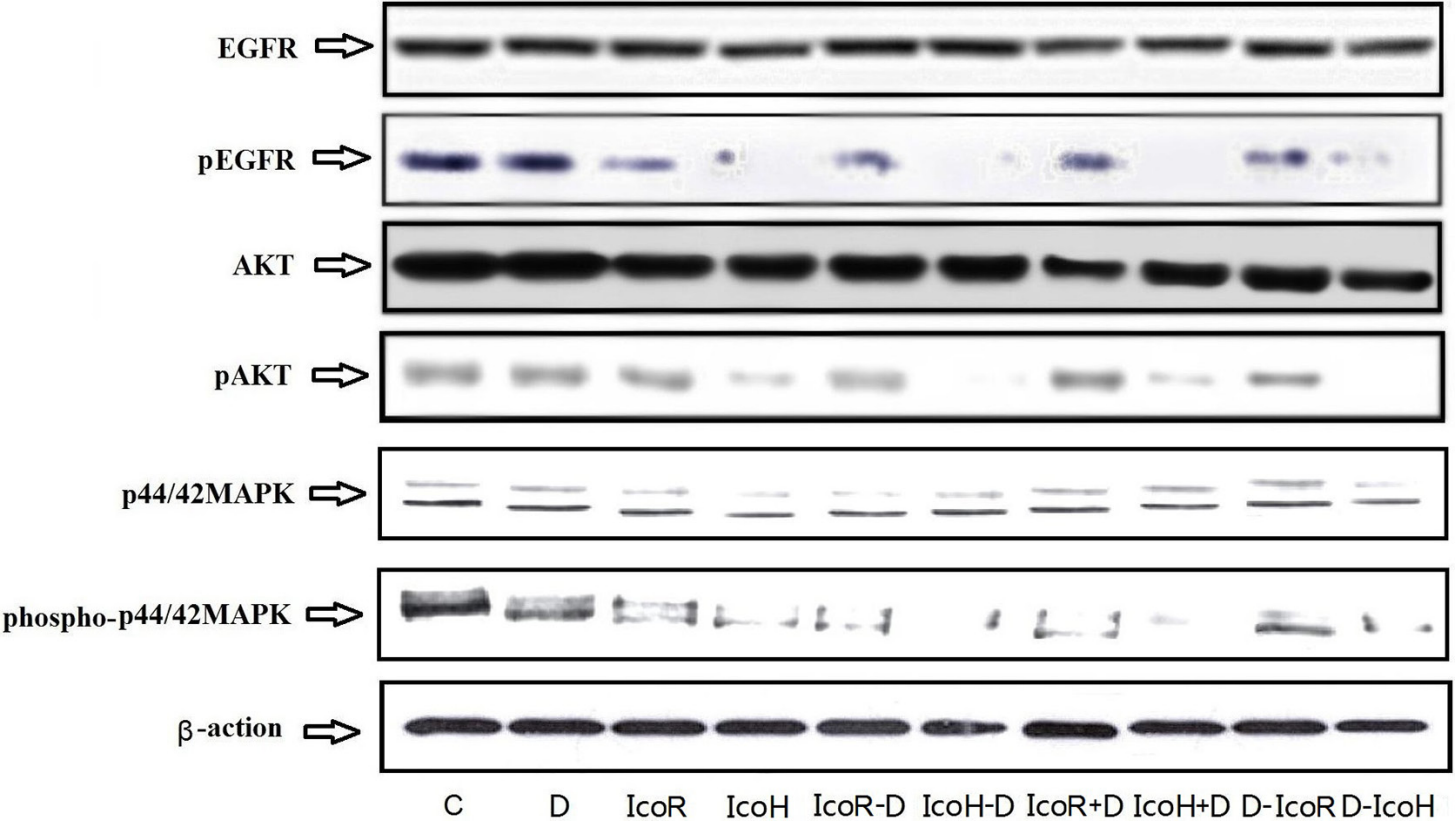
Expression and phosphorylation of tumor-related proteins in tumor xenografts The mice were subcutaneously injected with NSCLC A549 cells and when tumor xenografts reached 5–6 mm^3^, the mice received different treatments for up to 6 weeks. After the experiments, tumor xenografts were resected and subjected to protein extraction and Western blot analysis of EGFR, MAPK, and Akt protein level and phosphorylation. C, negative control group; D, docetaxel; IcoR, a regular icotinib dose; IcoH, a high icotinib dose; IcoH-D, a high icotinib dose and sequential docetaxel for 3 weeks; IcoR-D, a regular icotinib dose and sequential docetaxel for 3 weeks; IcoH+D, a high icotinib dose plus docetaxel for 3 weeks; IcoR+D, a regular icotinib dose plus docetaxel for 3 weeks; D-IcoR, docetaxel and sequential regular icotinib dose for 3 weeks; D-IcoH, docetaxel and sequential high icotinib dose for 3 weeks.

At the end of the experiments, we resected tumor xenografts and prepared frozen tissue sections for Western blot analysis. Our data showed that icotinib-containing treatment reduced phosphorylation of EGFR, MAPK, and Akt proteins (Figure 2), suggesting that the changed activity of these proteins was not sufficient to inhibit tumor growth (comparing Table 2 data with Figure 2). In contrast, docetaxel treatment did not affect activity of these proteins compared to the negative control group (Figure 2).

**Table 2:** Changes in microvessel density (mean ± SD) in wild-type EGFR NSCLC cells-generated xenografts after treatment of mice with icotinib, docetaxel, or their different combinations of order

Treatment	Microvessel density	% of control	*P* value
**Group A**			
Control	29.96 ± 1.92	Reference	
IcoR	28.05 ± 2.49	93.6	< 0.05^##^
IcoH	25.09 ± 2.41	83.7	< 0.05^#,##^
DTX	20.75 ± 1.96	69.2	< 0.05^#^
**Group B**			
IcoR+DTX	20.91 ± 1.45	69.7	< 0.05^#^
IcoR-DTX	21.83 ± 1.89	72.8	< 0.05^#^
DTX-IcoR	20.30 ± 1.67	67.7	< 0.05^#^
**Group C**			
IcoH+DTX	17.64 ± 2.40	58.8	< 0.05^#,##^
IcoH-DTX	17.45 ± 1.74	58.2	< 0.05^#,##^
DTX-IcoH	17.66 ± 1.91	58.9	< 0.05^#,##^

Abbreviations: Microvessel density was estimated by CD31 immunostaining in tissue xenografts.

Control, negative control; DTX, docetaxel; IcoR, a regular icotinib dose; IcoH, a high icotinib dose; IcoH-D, a high icotinib dose and sequential docetaxel for 3 weeks of treatment; IcoR-D, a regular icotinib dose and sequential docetaxel for 3 weeks; IcoH+D, a high icotinib dose plus docetaxel for 3 weeks; IcoR+D, a regular icotinib dose plus docetaxel for 3 weeks; D-IcoR, docetaxel and sequential regular icotinib dose for 3 weeks; D-IcoH, docetaxel and sequential high icotinib dose for 3 weeks; TGIR, tumor growth inhibition rate.

^#^*P* < 0.05 compared to the negative control; ^##^*P* < 0.05 compared to docetaxel.

### Effect of icotinib and docetaxel treatment on regulation of microvessel density in tumor cell xenografts

**Figure 3:**
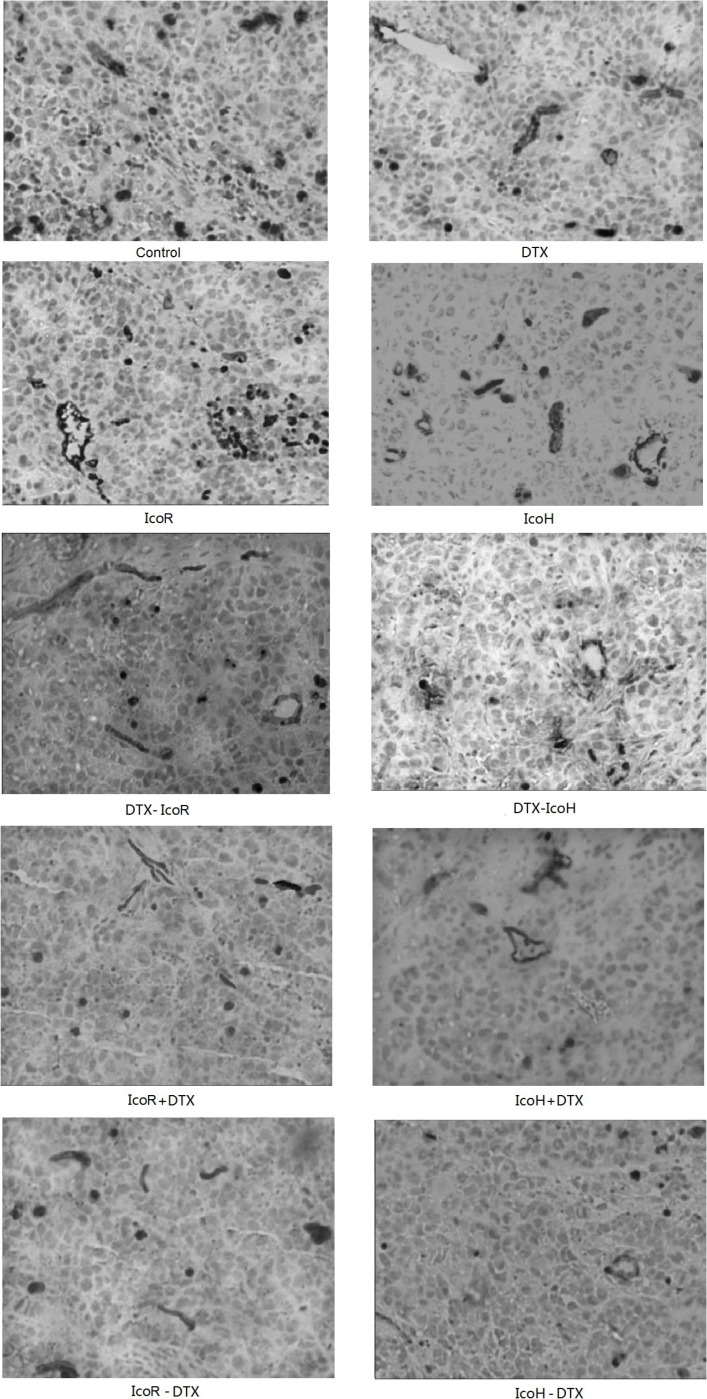
Immunohistochemical detection of CD34 expression in tumor xenografts The mice were subcutaneously injected with NSCLC A549 cells and when tumor xenografts reached 5–6 mm^3^, the mice were received different treatments for up to 6 weeks. At the end of experiments, tumor xenografts were resected and subjected to immunohistochemical analysis of CD34 level. Control, negative control; DTX, docetaxel; IcoR, a regular icotinib dose; IcoH, a high icotinib dose; IcoH-D, a high icotinib dose and sequential docetaxel for 3 weeks; IcoR-D, a regular icotinib dose and sequential docetaxel for 3 weeks; IcoH+D, a high icotinib dose plus docetaxel for 3 weeks; IcoR+D, a regular icotinib dose plus docetaxel for 3 weeks; D-IcoR, docetaxel and sequential regular icotinib dose for 3 weeks; D-IcoH, docetaxel and sequential high icotinib dose for 3 weeks.

We then immunostained CD34 protein in these tumor xenografts for calculation of microvessel density and found that docetaxel alone could significantly reduce microvessel density in tumor xenografts compared to the negative control group (29.9 ± 1.92 vs. 20.75 ± 1.96, *P* < 0.05), while the high dose of icotinib alone only mildly reduced microvessel density in tumor xenografts (29.9 ± 1.92 vs. 25.09 ± 2.41, *P* < 0.05; Table 2 and Figure 3). The regular dose of icotinib plus or sequential docetaxel treatment did not show any additive effects on inhibition of microvessel density in tumor xenografts.

In contrast, the high dose of icotinib alone or with sequential docetaxel treatment showed at least an additive effect on inhibition of microvessel density in tumor xenografts (Table 2 and Figure 3). These data suggest that the additive effect of icotinib (1200 mg/kg) plus sequential docetaxel on suppression of NSCLC cell nude mouse xenograft growth may be caused by reduction of the microvessel density in tumor xenografts.

## DISCUSSION

Icotinib hydrochloride is a novel EGFR-TKI, which can reversibly bind to the EGFR protein ATP binding site and in turn prevent the EGFR signal transduction cascade. Therefore, icotinib is able to inhibit EGFR-led cell growth signaling [24–28]. A previous phase III clinical trial of 400 advanced NSCLC patients revealed that progression-free survival of icotinib-treated patients was similar to those treated with gefitinib, with icotinib having less adverse events than gefitinib [24]. Usually, EGFR-TKIs are used to treat patients with EGFR mutated or amplified NSCLC, and in the current study, we explored icotinib antitumor activity after it was combined with (or sequential to) docetaxel in growth of wild-type EGFR A549 cells-generated nude mouse xenografts.

Our (unpublished) data showed that icotinib could be useful in the inhibition of EGFR activity in A549 cells expressed EGFR protein in tumor xenografts. Docetaxel is a third-generation chemotherapeutic cytotoxic drug derived from taxane compounds that can inhibit cell division; thus, it is frequently used to treat NSCLC, breast cancer, ovarian cancer, head and neck cancer, gastric cancer, and hormone-refractory prostate cancer [29] to increased median and disease-free survival of these patients [30]. In the current study, we selected docetaxel for combination or sequential treatment with icotinib in a nude mouse model of NSCLC cell xenografts.

Saigal et al. (2008) reported *in vitro* data on different head and neck squamous cell carcinoma cell lines treated with different orders and doses of docetaxel combined with EGFR-TKI [31]. They showed that erlotinib synergistically inhibited tumor cell growth in an erlotinib pre-treatment group, whereas the docetaxel pre-treatment group experienced no synergistic effect, suggesting that EGFR-TKI used before the cytotoxic drug had a synergistic effect on inhibition of tumor cell growth, but not thereafter in a MDA1986 cell line [31]. Our current study further confirmed this finding.

In our current study, we selected regular and high doses of icotinib combined with or sequential to docetaxel to explore antitumor activity in tumor xenograft model in nude mice. Selected dosages were based on our preliminary *in vivo* experiments using 600 mg, 1200 mg, and 1800 mg data when we discovered that 1200 and 1800 mg had similar antitumor effects, while 600 mg had lesser effects; thus, we selected a low dose of 60 mg/kg and a high dose of 1200 mg/kg for the present study.

Our data showed that the antitumor effect of the high dose of icotinib for 2 days before docetaxel was significantly better than other treatment groups in terms of tumor growth inhibition rate and tumor size, while the high dose of icotinib combined with docetaxel was the second best in terms of tumor growth inhibition rate and tumor size; the antitumor effect of docetaxel alone or combination with the regular dose of icotinib was similar (*P* > 0.05). In a previous study, Solit et al. (2005) performed a similar study of wild-type EGFR breast cancer cells-generated nude mouse xenografts [32] and showed that compared to a 5-day continuous administration of paclitaxel, gefitinib treatment at a dose of 65 mg/kg for 2 days before paclitaxel had obvious antitumor activity and that a 150 mg/kg gefitinib had the best tumor inhibition, suggesting the higher dose of EGFR-TKI pretreatment resulted in a better antitumor activity [32], while our current study showed the similar data and therefore confirmed their data. Furthermore, Boehrer et al. (2008) showed that erlotinib was able to inhibit growth of acute myeloid leukemia cell xenografts [33] and explained the non-target effect of erlotinib, i.e., erlotinib-induced apoptosis and phenotypic differentiation of acute myeloid leukemia cells were not through erlotinib-inhibited EGFR tyrosine kinase receptor pathway, but could be due to inhibition of MAPK and Akt gene pathways [33].

In our current study, we found that icotinib-containing treatment was able to suppress activities of EGFR, MAPK, and Akt proteins; it was not total inhibition of protein phosphorylation, but reduction of tumor xenograft volume, and size may not be through suppression of these protein activities. Instead, we found that reduction of microvessel density after the high dose icotinib-containing treatment may be responsible for reduced tumor xenograft volume and size, although we do know the exact underlying mechanism by which the high dose of icotinib-containing treatment affects microvessel density. Further study will focus on neoangiogenesis-related genes and pathway after high-dose icotinib-containing treatment.

Moasser et al. (2007), in their study of EGFR-TKIs in regulation of tumor vascular functions [34], showed that paclitaxel combined with high-dose gefitinib was better than paclitaxel or gefitinib alone as well as paclitaxel combined a regular-dose gefitinib. They also found that vascular perfusion of human BT474 breast cancer xenografts in nude mice was poor in the control group, but moderate improvements in the gefitinib group appeared because of changed tumor vascular structure, thus they suggested that TKI treatment normalized the tumor microenvironment and benefited the cytotoxic drugs. The endothelial-transferring constant (Kps) was transient but obviously decreased in the high-dose gefitinib pre-group on the 3rd day. On the 8th day of the experiments, the Kps recovered to its initial level. However, this change did not occur in other groups. Similar changes also occurred in the tumor fractional plasma volume (fPV), which was increased on the 3rd day and returned back on the 8th day.

In our current study, we found that CD34 expression was significantly reduced in the high-dose icotinib with docetaxel combination groups compared to other experimental treatment groups. The changes in Kps and fPV in Moasser's data were temporary, as although a high dose of EGFR-TKI to improve tumor vascular hemodynamics is immediate, the histology may not change in the short period of time. This may explain why there was no statistically significant difference among the three different sequence treatment groups that took high-dose icotinib and docetaxel. We speculate that high-dose icotinib could improve tumor vascular hemodynamics thereby increasing the effect of cytotoxic drugs on wild-type *EGFR* NSCLC cell xenografts.

## MATERIALS AND METHODS

### Cell lines and culture

A human NSCLC cell-line A549 was obtained from Central Laboratory of Shandong Cancer Hospital and Institute (Jinan, Shandong, China) and maintained in a mixture of Dulbecco's modified Eagle's medium (DMEM) and F-12 or RPMI (1:1) supplemented with 10% newborn bovine serum and penicillin G (100k U/L) at 37°C in 5% CO_2_ and 95% air atmosphere.

### Animal experiments

This study was approved by the Institutional Animal Care and Use Committee (IACUC) of Shandong Cancer Hospital and Institute (Jinan, China). Four to 6-week-old nu/nu athymic female mice (19–22 g) were purchased from Biobase Company (Jinan, China) and maintained in ventilated cages. A549 cells (1 × 10^7^ cell per mouse/injection) were inoculated into the left flanks of 80 nude mice to establish the lung cancer cell mouse xenograft model.

After tumors reached to a size of 5 to 6 mm in diameter, these mice were randomly divided into the negative control (PBS), IcoR, IcoH, DTX, IcoR+DTX, IcoH+DTX, IcoR-DTX, IcoH-DTX, DTX-IcoR, and DTX-IcoH groups for treatment accordingly for three weeks. The regular icotinib (Beta Pharma, Inc., Shanghai, China) dose was 60 mg/kg, while the high dose was 1200 mg/kg; both doses were administered orally.

Docetaxel (Qilu-pharma, Jinan, China) was administered by intravenous injection at a dose of 5 mg/kg. Single and combination drug treatments were given to the mice for 3 weeks (see details in Table 1) and tumor cell xenograft formation and growth were monitored twice a week using a caliper.

Tumor growth inhibition rate was calculated at the end of experiments using the formula: [(L × W^2^)/2] to calculate tumor xenograft volume (Vt). Tumor growth inhibition (TGI) was calculated using the formula TGI = 1-[(T -T_0_) / (C-C_0_)] × 100 where T is the tumor volume after treatment, T_0_ is the tumor volume at the beginning of the treatment, C is the tumor volume of negative control at the end of the experiment, and C_0_ is the tumor volume at the beginning of the experiment.

At the end of experiments, all tumor cell xenografts were resected and subjected to Western blot analysis of gene expression or immunohistochemical analysis of CD31 level to estimate microvessel density (see below for details).

### Protein extraction and Western blot

Tumor cell xenografts were cut into 1 × 1 mm pieces on ice and grinded and then scraped into 1 mL of the lysis buffer containing 1% Triton-100, 0.1 mM phenylmethylsulfonyl fluoride (PMSF), 1 μM pepstatin, 0.5 mg/mL, leupeptin, 0.3 μM aprotinin, 50 mM Tris-HCl (pH 8.0), and 150 mM NaCl. After being lysed on ice for 30 min, the protein samples were centrifuged at 15,000 rpm for 20 min, the supernatants transferred into new tubes, and protein concentration was assayed using the BCA protein assay kit (CWBIO, Beijing, China). For Western blotting, these protein samples were subjected to sodium dodecyl sulfate-polyacrylamide gel electrophoresis (SDS-PAGE) using 10–15% of SDS-PAGE gels and transferred onto polyvinyldine diflouride membranes (Millipore, Billerica, MA, USA). The membranes were then blocked with 5% bovine serum albumin (BSA) and incubated for 18 h at 4°C with a monoclonal anti-EGFR, anti-phospho-EGFR, anti-MAPK, anti-phospho-MAPK, anti-Akt, anti-phospho-Akt, or anti-β-actin antibody (CWBIO). The next day, the membranes were washed for three times with Tris-based saline-0.1% Tween 20 (TBS-T) and then further incubated for 2 h at room temperature with an HRP-conjugated secondary antibody (CWBIO). Immunoreactive protein bands were further visualized by using the enhanced chemiluminescence (ECL) system (Pierce, Rockford, IL, USA) and exposed to X-ray films.

### Immunohistochemistry

Frozen tumor cell xenografts were sliced into 5-μM-thick tissue sections and fixed in acetone at –20°C for 10 min and then washed with PBS. The potential endogenous peroxidase activity was blocked with 0.3% hydrogen peroxide (H_2_O_2_) in methanol for 30 min at room temperature and the sections were then rinsed with PBS three times and incubated at 4°C overnight with a primary antibody (anti-mouse CD34). On the next day, sections were washed with PBS three times and then further incubated with a secondary antibody from the ABC kit (CWBIO) for 30 min at room temperature. The color reaction was performed using diaminobenzidine solution (CWBIO) briefly at room temperature and then briefly counterstained with a hematoxylin solution.

The histological sections were then independently reviewed and scored for CD34 expression under an Olympus microscope (Olympus, Shanghai, China) by NT, QZ, and SF. Any discrepancies were resolved by their consensus—i.e., they selected the three most positively stained fields under a 10× magnification and counted CD34 positivity (intensity of staining and percentage of cells stained) at a magnification of 200× to estimate the microvessel density for each immunostained section (a CD34-positive single cell or group of cells was counted as a microvessel; each microvessel had a clear boundary from other microvessels or tumor nests). The mean ± SD density of each xenograft was summarized.

### Statistical analysis

Statistical analysis was performed using SPSS v17 software (SPSS Inc., Chicago, IL, USA). All data were expressed as mean ± SD and statistical analyses were performed using *t*-test or Mann–Whitney *U* test, while the measurement data were compared with single factor analysis of variance. All *p*-values were two-sided and *p* ≤ 0.05 was considered statistically significant.

## CONCLUSIONS

Our current study revealed that the high-dose icotinib and sequential docetaxel treatment (IcoH-DTX) had an additive effect on suppressing the growth of wild-type EGFR A549 cells-generated nude mouse xenografts. Future studies using randomized controlled clinical trials with more robust data are needed to confirm our current observations and finding.
